# Supplementary material for: Control of self-assembly pathways toward conglomerate and racemic supramolecular polymers
Source: Nat Commun. 2020 Oct 29;11:5460. doi: 10.1038/s41467-020-19189-8 (PMC7596528; doi:10.1038/s41467-020-19189-8)
Supplement: Supplementary file 1 — Supplementary Information [file 41467_2020_19189_MOESM1_ESM.pdf]

Supplementary Information

Control of self-assembly pathways toward conglomerate and racemic supramolecular polymers

Marius Wehner,[†, #] Merle Insa Silja Röhr,[†] Vladimir Stepanenko,[#] Frank Würthner[†,#,*]

[†] Center for Nanosystems Chemistry & Bavarian Polymer Institute, Universität Würzburg, Theodor-Boveri-Weg, 97074 Würzburg, Germany

[#] Institut für Organische Chemie, Universität Würzburg, Am Hubland, 97074 Würzburg, Germany

1. Supplementary Methods

General

All chemicals and solvents were purchased from commercial suppliers and used without further purification. The ^{1}H and ^{13}C-NMR spectra were recorded with Bruker Avance III HD 400 or 600 MHz spectrometers and calibrated against the residual proton signal or natural abundance carbon resonance of the used deuterated solvent from tetramethylsilane (TMS) as the internal standard. The chemical shifts δ are indicated in ppm and the coupling constants J in Hz. The multiplicities are given at center of the respective signal as s (singlet), d (doublet), dd (doublet of doublets), t (triplet), q (quartet) and m (multiplet). The MALDI-TOF mass spectra were measured with a Bruker Daltonics autoflex II LRF by using *trans*-2-[3-(4-*tert*-butylphenyl)-2-methyl-2-propenylidene]malononitrile (DCTB) as a matrix. The high-resolution mass spectra (HRMS) were measured by electrospray ionization (ESI) with a microTOF Focus spectrometer from Bruker Daltonics.

Supplementary synthesis and characterization

The target compound ***(S,S)*-PBI** was synthesized according to a previously reported synthetic route[1] as outlined in Supplementary Fig. 1. 3,4,5-Tris(dodecyloxy)benzoic acid (**1**)[2] and (*S*)-*tert*-butyl (1-aminopropan-2-yl)carbamate (**2**)[3-5] were synthesized according to literature known procedures.

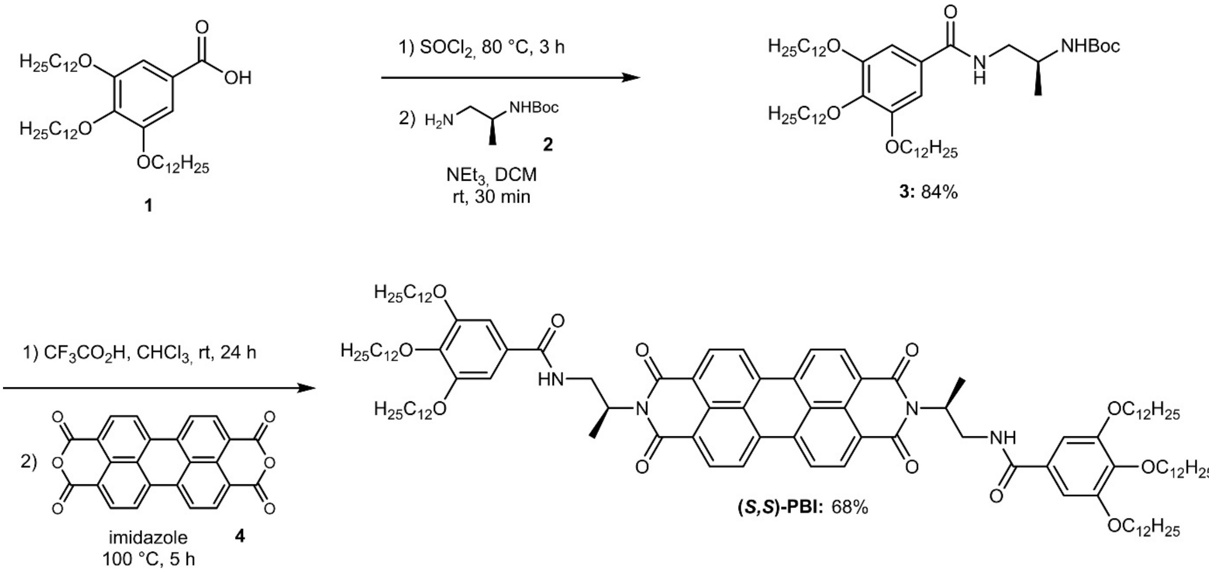

Supplementary Figure 1 | Synthesis of *(S,S)*-PBI. Synthesis of ***(S,S)*-PBI** starting from literature known 3,4,5-trisdodecylbenzoic acid (**1**) (Boc = *tert*-butyloxycarbonyl, DCM = dichloromethane).

(*S*)-*tert*-Butyl (1-(3,4,5-tris(dodecyloxy)benzamido)propan-2-yl)carbamate (3)

A mixture of 3,4,5-tris(dodecyloxy)benzoic acid (**1**) (697 mg, 1.03 mmol) and thionyl chloride (1.5 mL) was stirred at 80 °C for 3 h under nitrogen atmosphere. Excess thionyl chloride was removed at 50 °C under reduced pressure and the colorless solid was dissolved in 10 mL dry dichloromethane (DCM). Subsequently, the solution was added dropwise to a solution of **2** (150 mg, 860 µmol) and NEt_3 (1 mL) in 15 mL dry DCM. After stirring the reaction mixture at room temperature for 30 min the solvent was removed under reduced pressure. The crude product was purified by column chromatography (SiO_2; DCM/MeOH 99.75:0.25). Rose solid (602 mg, 720 µmol, 84%). Mp. 93 – 94 °C. ^{1}H-NMR (400 MHz, $CDCl_3$): δ = 7.41 (br, 1H), 7.05 (s, 2H), 4.67 (br, 1H), 4.02 (t, ^3J = 6.6 Hz, 4H), 3.98 (t, ^3J = 6.6 Hz, 2H), 3.90 (m, 1H), 3.51 (m, 1H), 3.36 (m, 1H), 1.84-1.69 (m, 6H), 1.50-1.42 (m, 6H), 1.40 (s, 9H), 1.36-1.21 (m, 51H), 0.88 (m, 9H). ^{13}C-NMR (100 MHz, $CDCl_3$): δ = 167.4, 157.2, 153.1, 141.0, 129.1, 105.6, 80.1, 77.3, 73.5, 69.3, 48.4, 46.8, 32.0, 30.4, 29.9, 29.8, 29.7, 29.5, 28.4, 26.2, 22.8, 18.9, 14.2. MS (MALDI, positive mode, DCTB in $CHCl_3$): *m/z* calculated for $C_{51}H_{94}N_2O_6$ $[M]^+$: 830.711, found: 830.700.

(*S*,*S*)-*N*,*N*'-di[3,4,5-tris(dodecyloxy)benzoylamino-1-methylethyl]-perylene-3,4:9,10-tetracarboxylic acid bisimide ((*S*,*S*)-PBI)

To a solution of **3** (250 mg, 300 µmol) in 5 mL $CHCl_3$, trifluoroacetic acid (0.1 mL) was added dropwise. The reaction mixture was stirred at room temperature for 24 h. After washing with aqueous $NaHCO_3$ (3 × 15 mL) the solvent was evaporated under reduced pressure. The resulting rose solid (210 mg) and perylene-3,4:9,10-tetracarboxylic acid bisanhydride (**4**) (49.0 mg, 120 µmol) were mixed in 3 g of imidazole. The reaction mixture was stirred under nitrogen atmosphere at 100 °C for 5 h. After cooling to room temperature, the mixture was dissolved in $CHCl_3$ (60 mL) and washed with 1N HCl (3 × 60 mL) and aqueous $NaHCO_3$ (60 mL). After evaporation of the solvent under reduced pressure, the crude product was purified by column chromatography (SiO_2; toluene/$CHCl_3$ 10:90) and preparative thin layer chromatography (SiO_2; $CHCl_3$). Red solid (149 mg, 81.9 µmol, 68% over 2 steps). Mp. 183 – 184 °C. ^{1}H-NMR (400 MHz, toluene-d_8, 365 K): δ = 8.43 (d, ^3J = 8.0 Hz, 4H), 7.80 (d, ^3J = 8.0 Hz, 4H), 7.25 (s, 4H), 6.66 (br, 2H), 5.71 (br, 2H), 4.55 (m, 2H), 4.12 (t, ^3J = 6.5 Hz, 4H), 3.90 (m, 8H), 3.68 (m, 2H), 1.84 (m, 4H), 1.72 (m, 8H), 1.65 (d, ^3J = 7.1 Hz, 6H), 1.61-1.21 (m, 108H), 0.94-0.87 (m, 18H). ^{13}C-NMR (150 MHz, toluene-d_8, 365 K): δ = 167.0, 164.2, 153.8, 142.9, 134.3, 131.1, 130.8, 129.6, 126.4, 124.7, 124.1, 122.9, 107.9, 73.6, 70.1, 50.3, 43.7, 32.2, 31.0, 30.1, 30.0, 29.8, 29.7, 26.6, 22.9, 20.7, 16.0, 14.0. MS (MALDI-TOF, positive mode, DCTB in $CHCl_3$): *m/z*

calculated for $C_{116}H_{177}N_4O_{12}$ $[M+H]^+$: 1818.336, found: 1818.350. HRMS (ESI-TOF, positive mode, MeCN/$CHCl_3$ 1:1): *m/z* calculated for $C_{116}H_{177}N_4O_{12}$ $[M+H]^+$: 1818.33575, found: 1818.33575.

2. Supplementary Figures

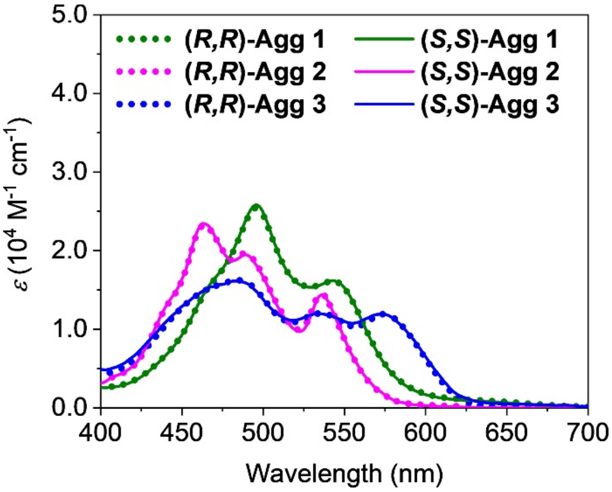

Supplementary Figure 2 | Absorption studies of (*R,R*)- and (*S,S*)-Agg 1–3. UV/vis absorption spectra of the three polymorphs of **(*R,R*)-** or **(*S,S*)-PBI** (c_T = 4.0 × 10^{-4} M, 298 K) in MCH/Tol (5:4, v/v) which are denoted as **(*R,R*)-Agg 1–3** or **(*S,S*)-Agg 1–3**, respectively.

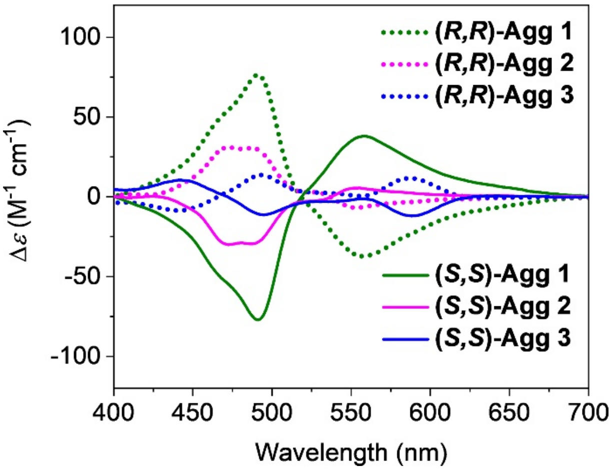

Supplementary Figure 3 | CD analyses of the three supramolecular polymorphs of (*R,R*)- and (*S,S*)-PBI. CD spectra of **(*R,R*)-Agg 1−3** and **(*S,S*)-Agg 1−3** (c_T = 4.0 × 10^{-4} M, 298 K) in MCH/Tol (5:4, v/v). The clear bisignate Cotton effects observed for **(*R,R*)-** and **(*S,S*)-Agg 1** correspond to *M*- and *P*-helical oligomers according to the exciton chirality method[6]. Notably, the CD spectra of the respective enantiomeric polymorphs **(*R,R*)-Agg 1−3** and **(*S,S*)-Agg 1−3** show mirror image behavior, i.e. **(*R,R*)-** and **(*S,S*)-PBI** form the same type of supramolecular polymorphs but with opposite helicities.

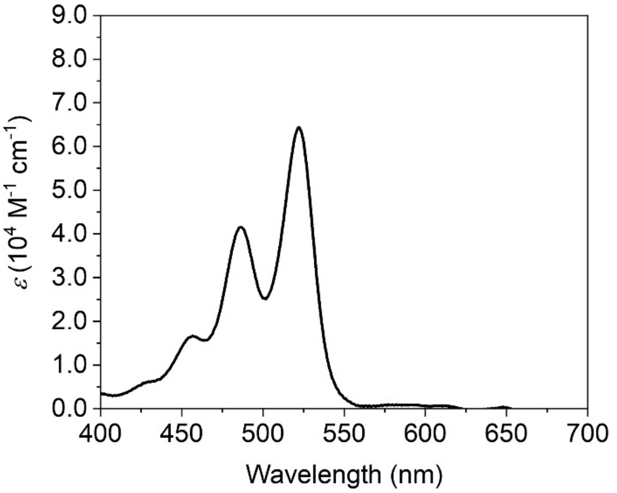

Supplementary Figure 4 | UV/vis-absorption spectrum of monomeric (*R*,*R*)-PBI. UV/vis-absorption spectrum of monomeric **(*R*,*R*)-PBI** ($c_T = 2.0 \times 10^{-6}$ M, 357 K) in MCH/Tol (5:4, v/v).

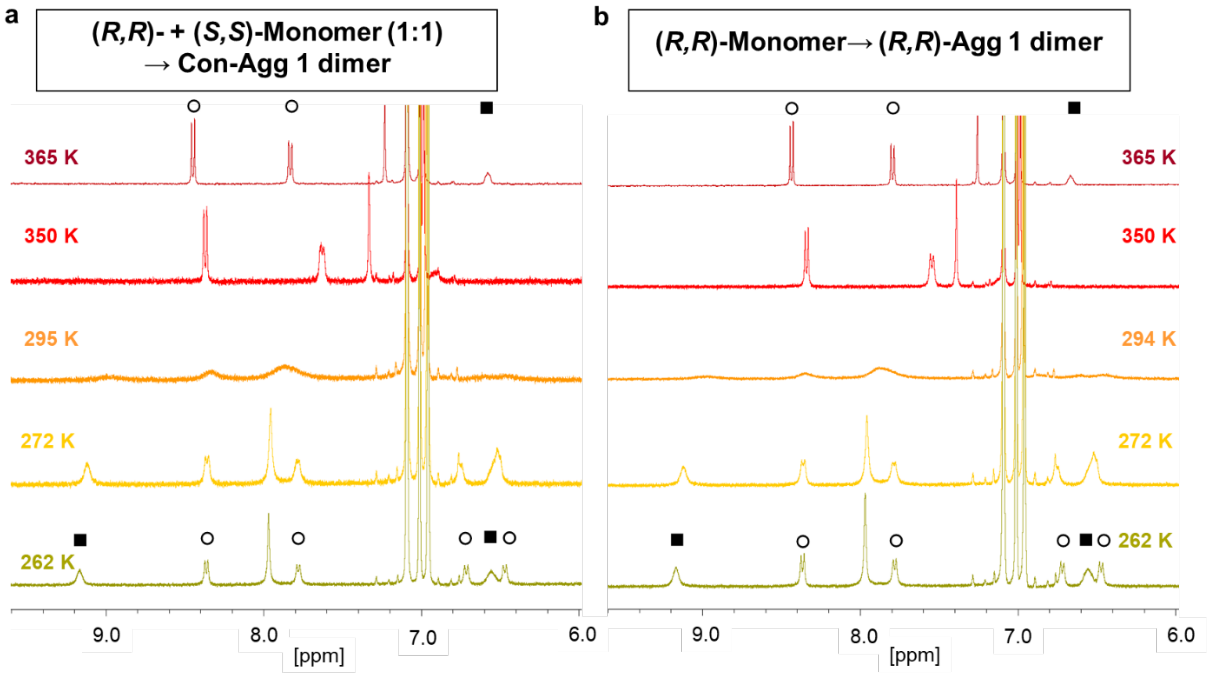

Supplementary Figure 5 | Variable temperature (VT) NMR studies. Partial VT-^{1}H-NMR spectra of the dimerization process of (**a**) **Con-Agg 1** and (**b**) **(*R*,*R*)-Agg 1**[1] in toluene-d^8 ($c_T = 4.0 \times 10^{-4}$ M). Upon decreasing temperature from 365 K to 262 K the racemic mixture of monomeric **(*R*,*R*)-** and **(*S*,*S*)-PBI** self-assembles into **Con-Agg 1** dimers (**a**) while monomeric **(*R*,*R*)-PBI** self-assembles into **(*R*,*R*)-Agg 1** (**b**).

Supplementary Note 1

Under the given experimental conditions, **(*R*,*R*)-PBI** as well as the racemic mixture of **(*R*,*R*)-PBI** and **(*S*,*S*)-PBI** exist as monomers at 365 K that are characterized by ^{1}H-NMR spectra with two doublets for the inner and outer perylene protons (open circles) and one peak for the N−H

protons (black squares). Upon decreasing the temperature from 365 to 262 K, the racemic mixture of monomeric **(*R*,*R*)-PBI** and **(*S*,*S*)-PBI** (Supplementary Fig. 5a) as well as monomeric **(*R*,*R*)-PBI** (Supplementary Fig. 5b) self-assemble into **Con-Agg 1** or **(*R*,*R*)-Agg 1** dimers, respectively, which are characterized by the same defined ^{1}H NMR spectra. The dimerization induces a splitting of the perylene protons into four doublets in both cases indicating that the perylene protons are not chemically equivalent within a dimer while they are in the respective monomeric species. Furthermore, a splitting of the N−H protons can be observed for both **(*R*,*R*)-** and **Con-Agg 1** dimers which may be explained, according to our previous studies[1], by the formation of two slightly different types of intermolecular hydrogen bonds between amide protons and imide oxygens of the adjacent PBIs within the respective dimers. At intermediate temperatures of 295 K or 294 K, NMR spectra with broad signals are observed for the racemic mixture (Supplementary Fig. 5a) and for enantiopure **(*R*,*R*)-PBI** (Supplementary Fig. 5b), respectively, which might result from a dynamic equilibrium between still present monomers and rather dynamic dimers which might not be interlocked in their final, more rigid conformation with close π-contacts and fourfold intermolecular hydrogen bonding as observed at 262 K.

Since both dimerization processes show the same VT-NMR spectra, it can be concluded that **Con-Agg 1** consists of equal amounts of **(*R*,*R*)-** and **(*S*,*S*)-Agg 1** which show the same ^{1}H-NMR spectra as **(*R*,*R*)-Agg 1** at the respective temperatures.

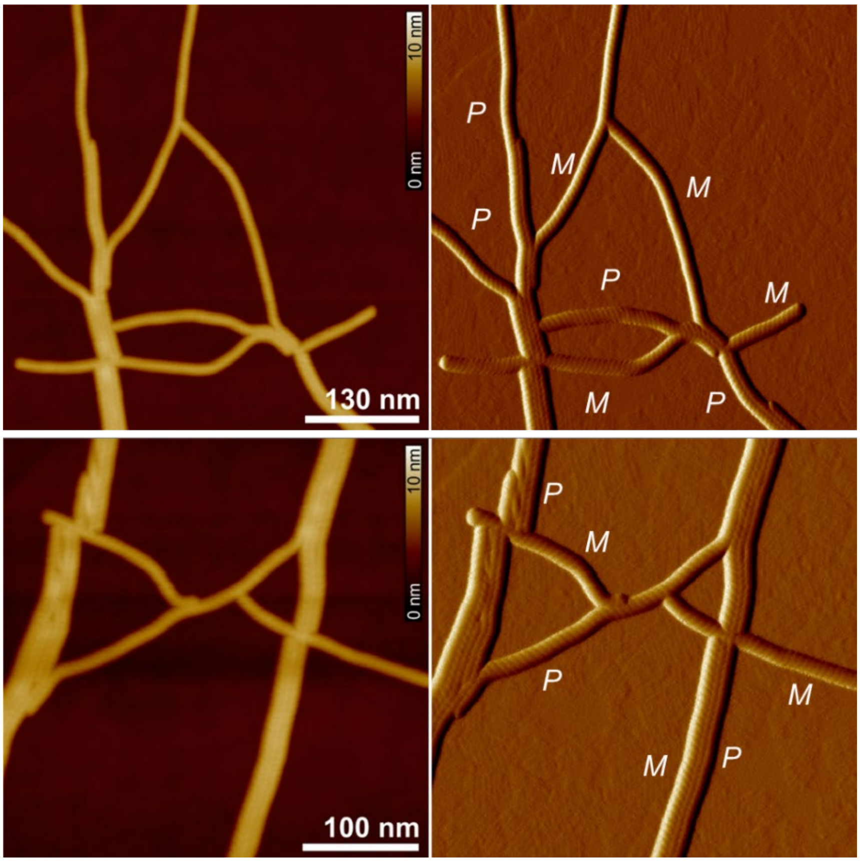

Supplementary Figure 6 | AFM studies of Con-Agg 2. AFM height and phase images of **Con-Agg 2** spin-coated on HOPG from MCH/Tol (5:4, v/v). *Z* scale is 10 nm. The helicities of the respective homochiral nanofibers of **Con-Agg 2** are indicated in the phase images.

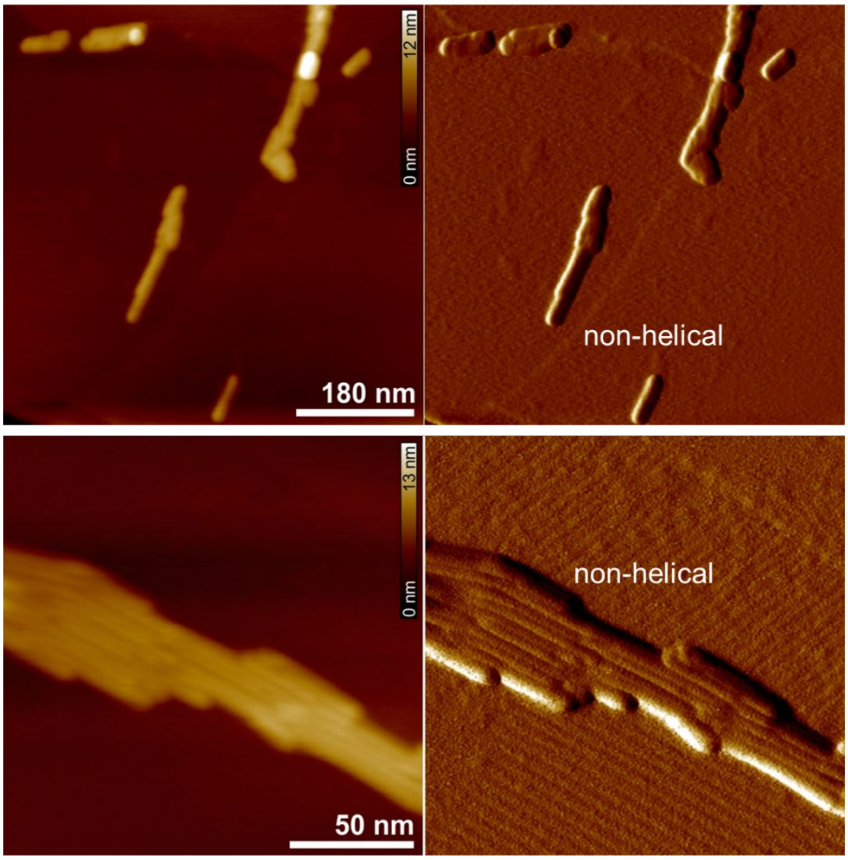

Supplementary Figure 7 | AFM studies of Rac-Agg 4. AFM height and phase images of **Rac-Agg 4** spin-coated on HOPG from MCH/Tol (5:4, v/v). *Z* scales are 12 and 13 nm, respectively. For **Rac-Agg 4** only non-helical nanorods were observed that further bundle into sheet-like structures.

Supplementary Table 1 | AFM analysis. Summary of morphology data for **Con-Agg 1**, **Con-Agg 2** and **Rac-Agg 4** obtained by AFM. Additionally, the respective data for **(*R*,*R*)-** and **(*S*,*S*)-Agg 1–3** is listed for comparison.

	Morphology	Helical pitch / nm	Diameter / nm	Length / nm
**Con-Agg 1**	Nanoparticles	–	3.8 ± 0.3	–
**Con-Agg 2**	Nanofibers	5.7 ± 0.2	4.5 ± 0.2	40 − 750
**Rac-Agg 4**	Nanorods	non-helical	4.1 ± 0.2	40 − 55
**(*R*,*R*)-Agg 1**	Nanoparticles	–	3.8 ± 0.3	–
**(*R*,*R*)-Agg 2**	Nanofibers	5.5 ± 0.3	4.7 ± 0.2	50 – 600
**(*R*,*R*)-Agg 3**	Nanofibers	16 ± 2	4.6 ± 0.2	45 – 450
**(*S*,*S*)-Agg 1**	Nanoparticles	–	3.8 ± 0.2	–
**(*S*,*S*)-Agg 2**	Nanofibers	5.4 ± 0.3	4.6 ± 0.2	60 – 800
**(*S*,*S*)-Agg 3**	Nanofibers	16 ± 2	4.5 ± 0.3	40 – 350

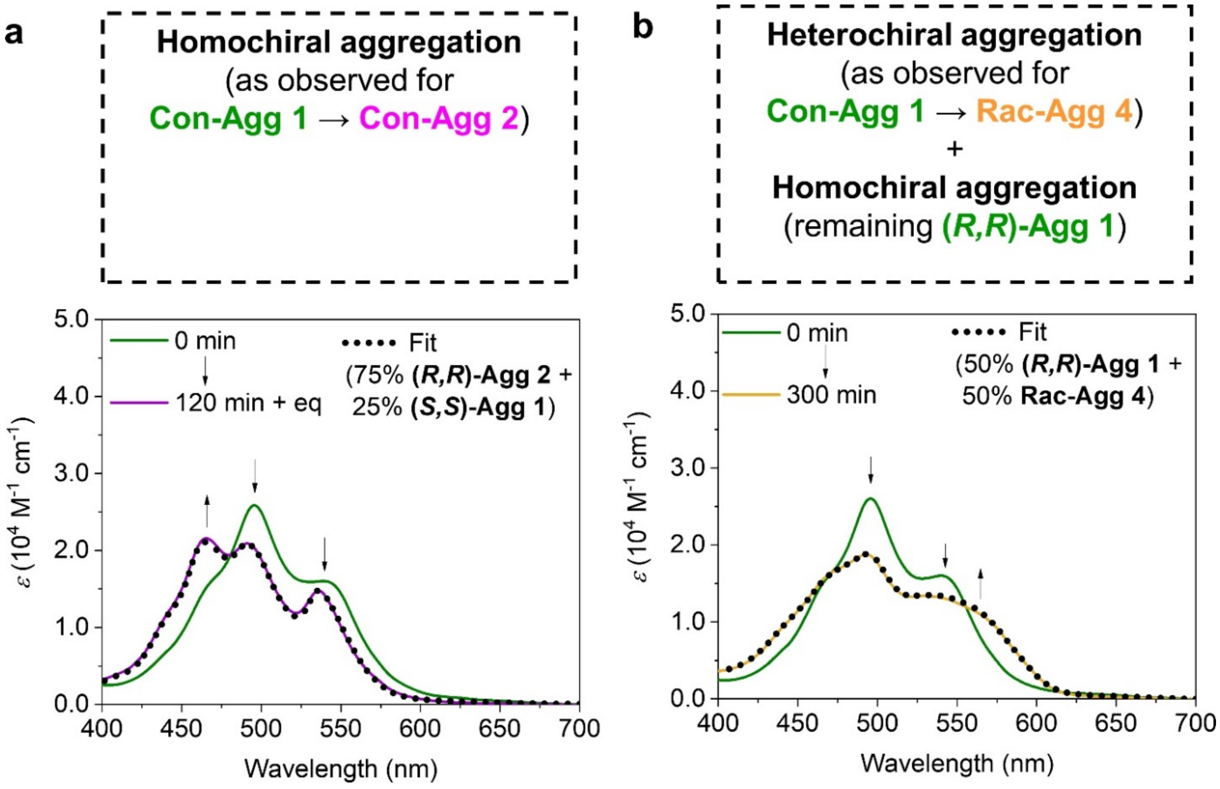

Supplementary Figure 8 | Ultrasound-induced transformations of enantiomerically imbalanced mixtures of (*R,R*)- and (*S,S*)-Agg 1. Initial and final UV/vis-absorption spectra of the ultrasound-induced transformations of mixtures of **(*R,R*)-** and **(*S,S*)-Agg 1** with enantiomeric excesses (*ee*) of **(*R,R*)-PBI** of *ee* = 50% in MCH/Tol (5:4, v/v) at 298 K (**a**: $c_T = 3.0 \times 10^{-4}$ M, ultrasonication at 293 – 298 K, **b**: $c_T = 4.0 \times 10^{-4}$ M, ultrasonication at 308 K). The respective final absorption spectra shown in (**a**) and (**b**) could be reconstructed from their constituting species as indicated in the respective graphs (black dots). "eq" denotes after equilibration at room temperature. Notably, the final absorption spectrum shown in (**b**) could not be reconstructed from the spectra of **(*R,R*)- / (*S,S*)-Agg 1** and **(*R,R*)-Agg 3**. According to our previous studies[1], the remaining **(*S,S*)-Agg 1** (**a**) or **(*R,R*)-Agg 1** (**b**) did not transform into any other species because the critical concentrations required for nucleation and transformation into **(*S,S*)-Agg 2** (**a**) or **(*R,R*)-Agg 3** (**b**) were not reached. Both outcomes further indicate that the majority-rules effect is not operative under these conditions.[7]

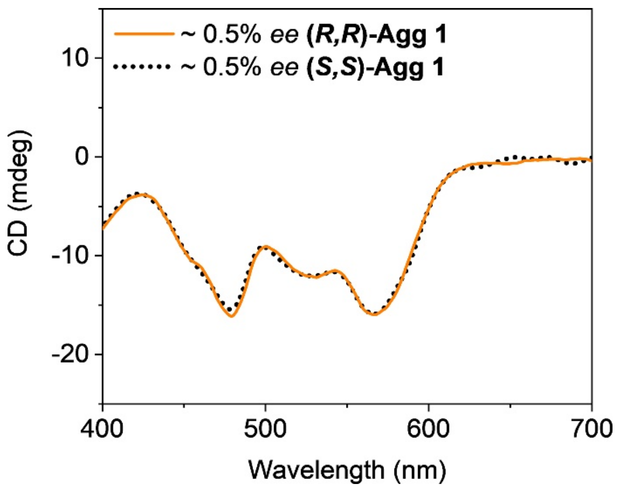

Supplementary Figure 9 | CD spectra of Rac-Agg 4 as a function of small enantiomeric excesses. CD spectra of "**Rac-Agg 4**" obtained after transformation of mixtures of ***(R,R)*****-** and ***(S,S)*****-Agg 1** with approximate enantiomeric excesses (*ee*) of ***(R,R)*****-** or ***(S,S)*****-PBI** of 0.5% in MCH/Tol (5:4, v/v) at 298 K ($c_T = 5.0 \times 10^{-4}$ M, ultrasonication at 308 K). The shape and CD signal intensities are independent of small *ee*'s.

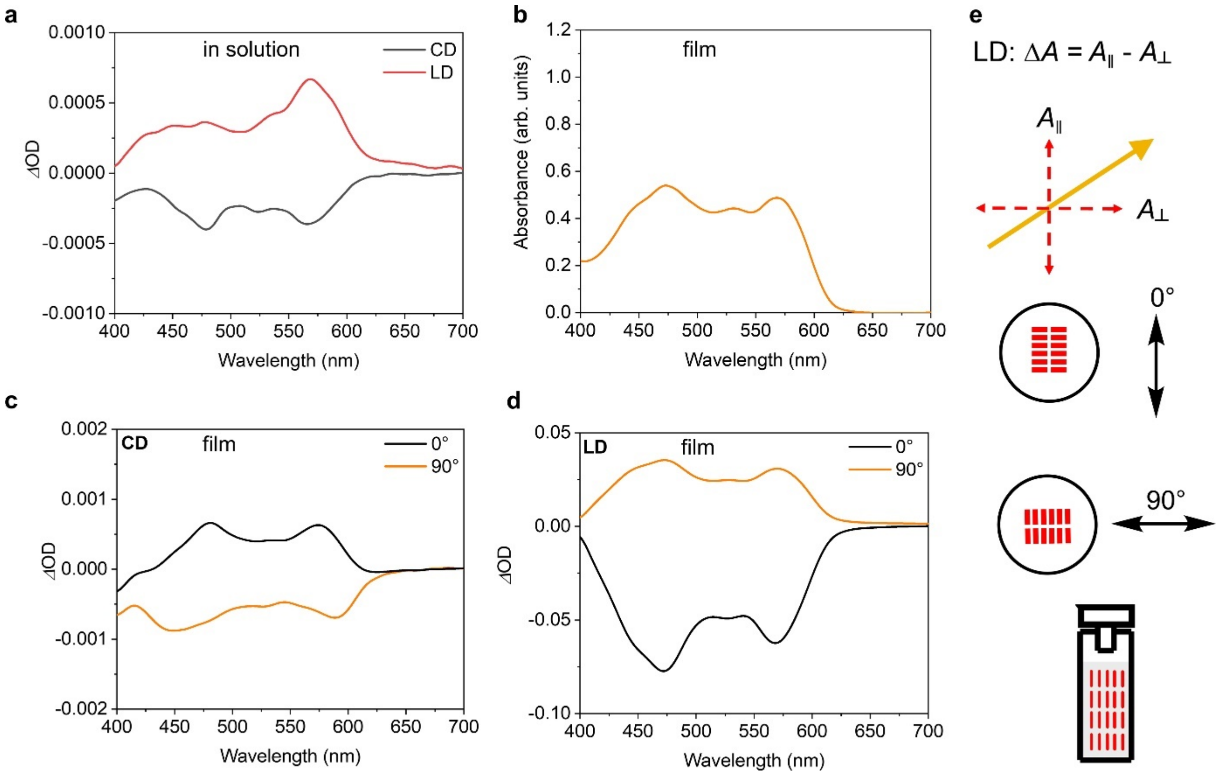

Supplementary Figure 10 | CD, LD and UV/vis studies of Rac-Agg 4. (**a**) CD and LD spectra of **Rac-Agg 4** in MCH/Tol (5:4, v/v) at 298 K ($c_T = 4.0 \times 10^{-4}$ M). (**b**) UV/vis-absorption spectrum of an aligned film (alignment as described in ref. [8]) of **Rac-Agg 4**. CD (**c**) and LD (**d**) spectra of the aligned film of **Rac-Agg 4** measured at 0° and 90°. (**e**) Schematic depiction of the LD effect which is defined as the difference in absorption of linearly polarized light parallel ($A_{\parallel}$) and perpendicular ($A_{\perp}$) to an orientation axis[9]. Furthermore, the orientation of the perylene bisimide (PBI) molecules within the aligned film and cuvette is indicated (red bars represent the PBIs' long and short axis).

Supplementary Note 2

The apparent CD signal of **Rac-Agg 4** (Fig. 1c main manuscript and Supplementary Fig. 10a) can be interpreted as a LD artefact (all CD instruments bear inherent optical imperfections leading to a contamination of circularly polarized light with linearly polarized light[8,10,11]) which results from the (partial) orientation of the molecules within the cuvette. In solution the observed LD signal is more intense than the CD signal (Supplementary Fig. 10a). In an aligned thin film no structural change of **Rac-Agg 4** upon alignment is observed (compare absorption spectra in solution (Fig. 1b main manuscript) and Supplementary Fig. 10b). The LD signal of the aligned film is more than 20 times larger than the respective CD signal of the aligned film (Supplementary Fig. 10c,d). The CD and LD results observed in solution can be qualitatively reproduced by measuring the aligned film in a 90° angle with respect to the alignment direction (Supplementary Fig. 10e) which proves that the respective PBI molecules are oriented vertically

with respect to their long molecular axis in the cuvette (Supplementary Fig. 10e, the measured $S_0 \rightarrow S_1$ electronic transition is polarized along the long molecular axis of the PBI molecules[12]).

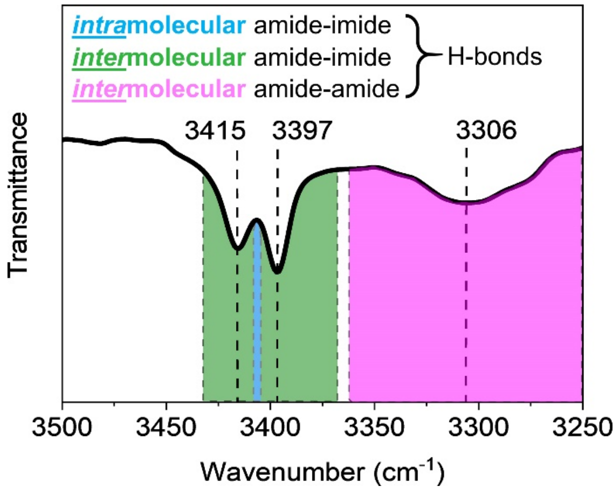

Supplementary Figure 11 | FT-IR study of Rac-Agg 4. FT-IR spectrum of the N−H stretching region of **Rac-Agg 4** in MCH/Tol (5:4, v/v) at 298 K ($c_T = 5.0 \times 10^{-4}$ M). The approximate spectral regions corresponding to the N−H stretching vibrations of intramolecularly amide-imide (blue), intermolecularly amide-imide (green) and intermolecularly amide-amide (magenta) hydrogen-bonded N−H groups are indicated based on previous results on the supramolecular polymorphs of **(*R*,*R*)-PBI** under the same experimental conditions[1].

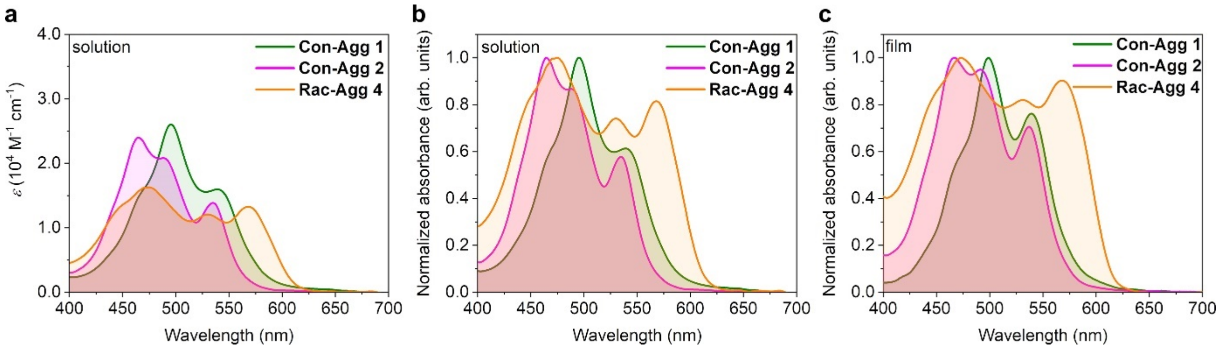

Supplementary Figure 12 | UV/vis studies of Con-Agg 1, Con-Agg 2 and Rac-Agg 4 in solution and solid state. UV/vis-absorption spectra (**a**) and normalized UV/Vis-absorption spectra (**b**) of **Con-Agg 1**, **Con-Agg 2** and **Rac-Agg 4** ($c_T = 5.0 \times 10^{-4}$ M) in MCH/Tol (5:4, v/v) at 298 K. (**c**) UV/vis-absorption spectra of thin films prepared by drop casting solutions of **Con-Agg 1**, **Con-Agg 2** or **Rac-Agg 4** on quarz plates. Notably, the thin-film absorption spectra resemble those obtained in solution.

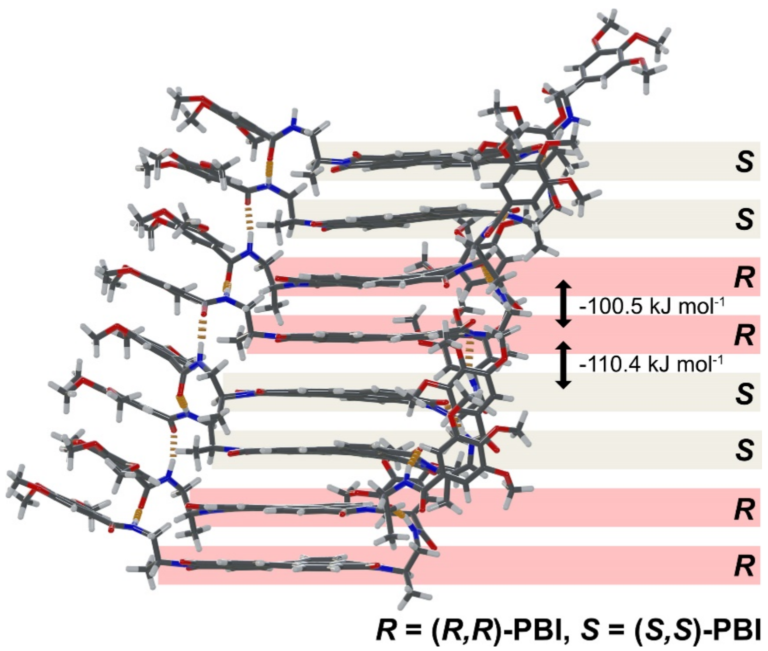

Supplementary Figure 13 | Quantum chemical calculations for Rac-Agg 4. Geometry-optimized octamer structure of **Rac-Agg 4** using PM6-D3H4[13-15] correction as implemented in the MOPAC software package[16]. *R* and *S* denote **(*R*,*R*)-** and **(*S*,*S*)-PBI**, respectively. The lower *RRSS*-tetramer stack is shown in the main manuscript. The calculated average gas-phase stabilization energies between the homochiral *RR* and heterochiral *RS* contacts are shown.

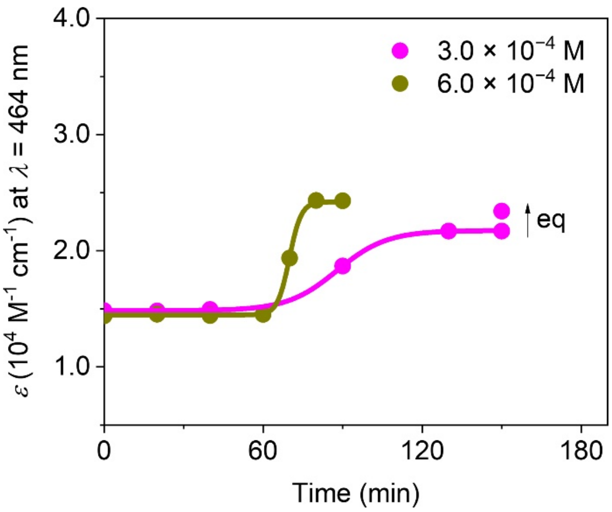

Supplementary Figure 14 | Kinetic analysis of the transformation of Con-Agg 1 into Con-Agg 2. Plot of the time-dependent ε values at λ = 464 nm derived from the respective absorption spectra at different concentrations for the ultrasound-induced transformation of **Con-Agg 1** → **Con-Agg 2**. Lines are set to guide the eye.

3. Supplementary Discussion

Elucidation of the thermodynamic parameters of Rac-Agg 4

The analysis of the thermodynamic parameters was conducted in analogy to ref. 1. For the analysis of the thermodynamic parameters the following considerations were made:

1) **Con-Agg 1** is a racemic mixture consisting of exclusively ***(R,R)*-** and ***(S,S)*-Agg 1** and virtually no hetero aggregates which was proven by UV/vis and NMR spectroscopy (Fig. 1b main manuscript and Supplementary Fig. 5).
2) The thermodynamic parameters of ***(S,S)*-Agg 1** are by definition the same as those of ***(R,R)*-Agg 1**[1] (Supplementary Table 2) due to their enantiomeric relation.
3) Since **Con-Agg 1** consists exclusively of ***(R,R)*-** and ***(S,S)*-Agg 1** and the thermodynamic parameters of ***(R,R)*-** and ***(S,S)*-Agg 1** are identical, the thermodynamic parameters of **Con-Agg 1** are accurately described by those determined for ***(R,R)*-Agg 1**[1] (Supplementary Table 2). In other words, **Con-Agg 1** can be treated as ***(R,R)*-Agg 1** with regard to its thermodynamics.

During the disassembly of **Rac-Agg 4** upon heating, the resultant monomers of ***(R,R)*-** and ***(S,S)*-PBI** reassemble into conglomerate **Con-Agg 1**. As previously reported for ***(R,R)*-Agg 1**[1], also **Con-Agg 1** is formed through an anticooperative process with preferential formation of even-sized aggregates due to the formation of tightly bound dimeric units with a higher dimerization constant K_2 of ***(R,R)*-** and ***(S,S)*-Agg 1** compared to the association constant K_E for further elongation (Supplementary Table 2)[1,17]. Thus, the overall monomer concentrations c_{Mono} at the critical temperatures of the disassembly processes of **Rac-Agg 4** (inset Fig. 5b main manuscript) were determined based on an anticooperative model that accounts for the preferential formation of even-numbered aggregates[17]. According to the aforementioned model, the known total concentration of molecules c_T can be expressed by the sum of the concentration of monomers c_{Mono}, molecules in even-numbered (second term) and odd-numbered (third term) aggregates at a certain temperature (Supplementary Equation 1).

$$c_T = c_{Mono} + \frac{2K_2c_{Mono}^2}{(1 - K_2K_Ec_{Mono}^2)^2} + \frac{K_2K_Ec_{Mono}^3(3 - K_2K_Ec_{Mono}^2)}{(1 - K_2K_Ec_{Mono}^2)^2} \qquad \text{Supplementary Equation 1}$$

For the determination of c_{Mono} at the respective critical temperature T_E (c_{Mono} (T_E)), the dimerization constant K_2 and the association constant for further elongation K_E of **Con-Agg 1**

(which are the same as those of **(*R*,*R*)-Agg 1**) at the critical temperatures T_E were put into Supplementary Equation 1[1].

After numerical determination of c_{Mono} (T_E), a van't Hoff plot (Fig. 5b main manuscript) was prepared according to Supplementary Equation 2 from which the standard enthalpy change ΔH^O and the standard entropy change ΔS^O upon aggregation were estimated:

$$\ln K(T_E) = \ln\left[\frac{1}{c_{Mono}(T_E)}\right] = \frac{-\Delta H^O}{RT_E} + \frac{\Delta S^O}{R} \quad \text{Supplementary Equation 2}$$

The Gibbs free energy change ΔG^O at 298 K was determined according to the Gibbs-Helmholtz equation (Supplementary Equation 3):

$$\Delta G^O = \Delta H^O - T\Delta S^O \quad \text{Supplementary Equation 3}$$

Supplementary Table 2 | Thermodynamic parameters of all supramolecular polymorphs. Thermodynamic parameters K_2, K_E, ΔH^O, ΔS^O and ΔG^O obtained for the supramolecular polymerization processes of **Con-Agg 1**, **Con-Agg 2**, **Rac-Agg 4** and of **(*R*,*R*)-** and **(*S*,*S*)-Agg 1−3** in MCH/Tol (5:4, v/v).

	**Con-Agg 1**<br><br>**(*R*,*R*)-Agg 1**<br>**(*S*,*S*)-Agg 1**<br>**Dimerization**	 <br><br><br>**Elongation**	**Con-Agg 2**<br><br>**(*R*,*R*)-Agg 2**<br>**(*S*,*S*)-Agg 2**	**(Con-Agg 3)**<br><br>**(*R*,*R*)-Agg 3**<br>**(*S*,*S*)-Agg 3**	**Rac-Agg 4**
**K_2 (298 K) (M^{-1})**	2.0×10^6				
**K_E (298 K) (M^{-1})**		1.8×10^5	7.5×10^5	1.0×10^6	2.6×10^6
**ΔH^O (kJ mol^{-1})**	−86.0	−86.0	−80.3	−96.3	−109
**ΔS^O (J mol^{-1} K^{-1})**	−168	−188	−157	−208	−243
**ΔG^O(298 K) (kJ mol^{-1})**	−35.9	−30.0	−33.5	−34.3	−36.6

The thermodynamic parameters of **(*R*,*R*)-Agg 1−3** were taken from ref. 1. Since **(*R*,*R*)-Agg 1−3** and **(*S*,*S*)-Agg 1−3** are enantiomers with the same absorption characteristics (Supplementary Fig. 2) the thermodynamic parameters of **(*S*,*S*)-Agg 1−3** are identical to those of **(*R*,*R*)-Agg 1−3**.

Con-Agg 1, **Con-Agg 2** and **Con-Agg 3** are conglomerates consisting of equal amounts of either **(*R*,*R*)-** and **(*S*,*S*)-Agg 1**, **(*R*,*R*)-** and **(*S*,*S*)-Agg 2** or equal amounts of **(*R*,*R*)-** and **(*S*,*S*)-**

Agg 3, respectively. Therefore, the thermodynamic parameters of **Con-Agg 1−3** are approximately identical to those of **(*R*,*R*)-Agg 1−3** under the assumption that no hetero contacts are formed which is suggested by VT ^{1}H-NMR spectroscopy (Supplementary Fig. 5). We note that **Con-Agg 3** can be only accessed by "manual" mixing of **(*R*,*R*)-** and **(*S*,*S*)-Agg 3** in a 1:1 ratio and is not formed upon ultrasonication of the racemic mixture of **(*R*,*R*)-** and **(*S*,*S*)-PBI**. Hence, it is written in parenthesis in Supplementary Table 2.

Next, the standard Gibbs free energies ΔG^{O} at 298 K of **Con-Agg 1−3** and **Rac-Agg 4** were used to calculate the racemic polymorph stabilities upon increasing aggregate size (Supplementary Fig. 15).

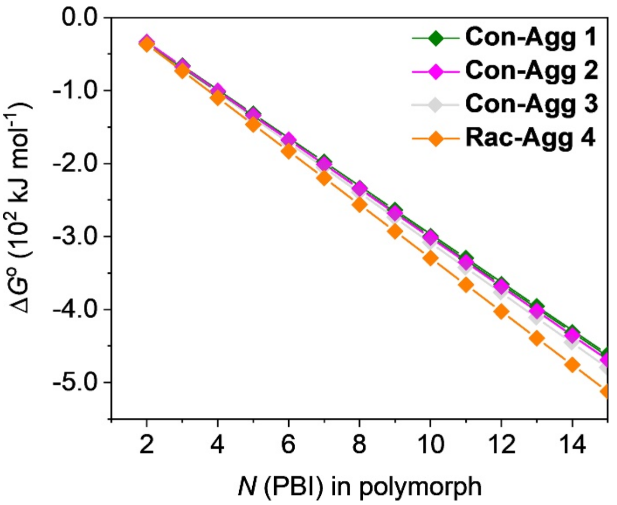

Supplementary Figure 15 | Comparison of polymorph stabilities of the racemic mixture. Changes in the standard Gibbs free energy ΔG^{O} of sections of stacks of the racemic mixture of **(*R*,*R*)-** and **(*S*,*S*)-PBI** consisting of *N* PBI molecules at 298 K. For comparison, the ΔG^{O} values of **Con-Agg 3** are also plotted although this species is not directly formed upon ultrasonication of the racemic mixture due to the formation of thermodynamically stable **Rac-Agg 4** (see discussion main text).

Further studies on the stabilities of the polymorphs

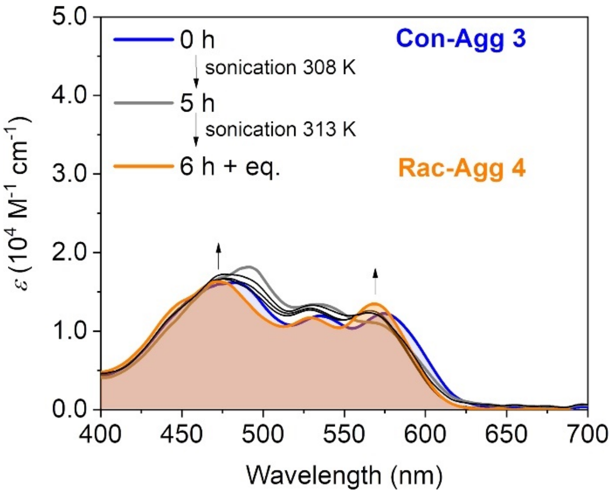

Supplementary Figure 16 | Ultrasound-induced transformation of manually prepared Con-Agg 3 into Rac-Agg 4. UV/vis-absorption spectra for the ultrasound-induced transformation of manually prepared **Con-Agg 3** into **Rac-Agg 4** upon ultrasonication at 308 K followed by ultrasonication at 313 K ($c_T = 4.0 \times 10^{-4}$ M) in MCH/Tol (5:4, v/v). After 6 h of ultrasonication at 313 K and equilibration at room temperature ("eq."), **Con-Agg 3** was completely transformed into **Rac-Agg 4** which proves that the racemic supramolecular polymer **Rac-Agg 4** is the thermodynamically most stable state of the racemic mixture.

Con-Agg 3 was manually prepared from **(*R,R*)-** and **(*S,S*)-Agg 3**. Therefore, enantiopure solutions of **(*R,R*)-Agg 1** or **(*S,S*)-Agg 1** were transformed into **(*R,R*)-** or **(*S,S*)-Agg 3**, respectively, and afterwards mixed in a 1:1 ratio. Due to its high kinetic stability, **Con-Agg 3** could not be transformed into **Rac-Agg 4** upon ultrasonication at 308 K for 5 h. However, sonication at an increased temperature of 313 K ("more thermodynamic conditions") led to the complete transformation of **Con-Agg 3** into **Rac-Agg 4** after 6 h and equilibration at room temperature which clearly proofs that **Rac-Agg 4** is the thermodynamically stable state of the racemic mixture and more stable than **Con-Agg 3** which is in accordance with the thermodynamic parameters presented in Supplementary Table 2. Our previous studies[1] showed that all polymorph transformations within enantiopure **(*R,R*)-PBI** occur on the level of **(*R,R*)-Agg 1** dimers. Together with the kinetic data of **Rac-Agg 4** formation (see main manuscript), these results indicate that the polymerization of **Con-Agg 3** into **Rac-Agg 4** most likely proceeds via the disassembly of **Con-Agg 3** into **Con-Agg 1** dimers (solid blue arrow, Fig. 6 main manuscript) and simultaneous transformation of these intermediates into **Rac-Agg 4** (orange arrow, Fig. 6 main manuscript). This polymerization pathway is further corroborated by the absence of isosbestic points in the UV/vis spectra for the transformation of **Con-Agg 3**

into **Rac-Agg 4** (Supplementary Fig. 16) which indicates that more than two species are in a dynamic equilibrium instead of a direct transformation of **Con-Agg 3** into **Rac-Agg 4**.

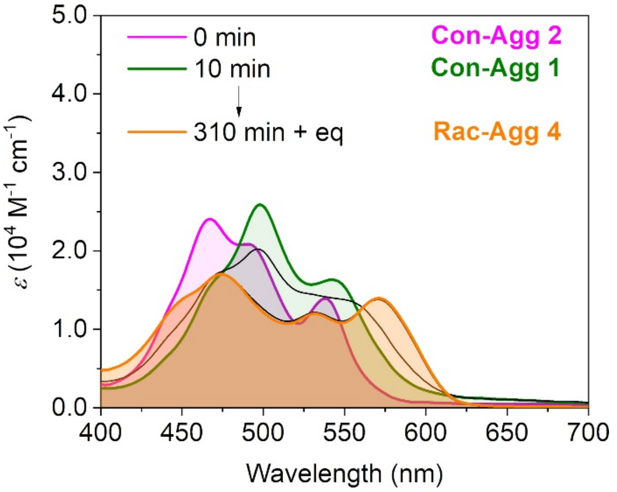

Supplementary Figure 17 | Ultrasound-induced transformation of Con-Agg 2 into Rac-Agg 4. UV/vis-absorption spectra for the transformation of **Con-Agg 2** → **Rac-Agg 4** with **Con-Agg 1** as an intermediate (c_T = 5.0 × 10^{-4} M, sonication at 308 K in MCH/Tol (5:4, v/v)) depending on the sonication period. "Eq" denotes equilibration at room temperature.

4. Supplementary References

1 Wehner, M. *et al.* Supramolecular Polymorphism in One-Dimensional Self-Assembly by Kinetic Pathway Control. *J. Am. Chem. Soc.* **141**, 6092-6107 (2019).

2 Tang, Y. *et al.* Giant Nanotubes Loaded with Artificial Peroxidase Centers: Self-Assembly of Supramolecular Amphiphiles as a Tool To Functionalize Nanotubes. *Angew. Chem. Int. Ed.* **49**, 3920-3924 (2010).

3 Hunt, T. *et al.* Discovery of a novel chemotype of potent human ENaC blockers using a bioisostere approach. Part 2: α-Branched quaternary amines. *Bioorg. Med. Chem. Lett.* **22**, 2877-2879 (2012).

4 Gobbini, M. *et al.* Novel Analogues of Istaroxime, a Potent Inhibitor of Na^{+},K^{+}-ATPase: Synthesis and Structure−Activity Relationship. *J. Med. Chem.* **51**, 4601-4608 (2008).

5 Nishiwaki, H., Nagaoka, H., Kuriyama, M., Yamauchi, S. & Shuto, Y. Affinity to the Nicotinic Acetylcholine Receptor and Insecticidal Activity of Chiral Imidacloprid Derivatives with a Methylated Imidazolidine Ring. *Biosci. Biotechnol. Biochem.* **75**, 780-782 (2011).

6 Harada, N. & Nakanishi, K. Exciton chirality method and its application to configurational and conformational studies of natural products. *Acc. Chem. Res.* **5**, 257-263 (1972).

7 Palmans, A. R. A. & Meijer, E. W. Amplification of Chirality in Dynamic Supramolecular Aggregates. *Angew. Chem. Int. Ed.* **46**, 8948-8968 (2007).

8 Wolffs, M. *et al.* Macroscopic Origin of Circular Dichroism Effects by Alignment of Self-Assembled Fibers in Solution. *Angew. Chem. Int. Ed.* **46**, 8203-8205 (2007).

9 Rodger, A., Dorrington, G. & Ang, D. L. Linear dichroism as a probe of molecular structure and interactions. *Analyst* **141**, 6490-6498 (2016).

10 Shindo, Y. & Ohmi, Y. Problems of CD spectrometers. 3. Critical comments on liquid crystal induced circular dichroism. *J. Am. Chem. Soc.* **107**, 91-97 (1985).

11 Shindo, Y. & Nishio, M. The effect of linear anisotropies on the CD spectrum: Is it true that the oriented polyvinyalcohol film has a magic chiral domain inducing optical activity in achiral molecules? *Biopolymers* **30**, 25-31 (1990).

12 Würthner, F. *et al.* Perylene Bisimide Dye Assemblies as Archetype Functional Supramolecular Materials. *Chem. Rev.* **116**, 962-1052 (2016).

13 Stewart, J. J. P. Optimization of parameters for semiempirical methods V: Modification of NDDO approximations and application to 70 elements. *J. Mol. Model.* **13**, 1173-1213 (2007).

14 Řezáč, J. & Hobza, P. Advanced Corrections of Hydrogen Bonding and Dispersion for Semiempirical Quantum Mechanical Methods. *J. Chem. Theory Comput.* **8**, 141-151 (2012).

15 Grimme, S., Antony, J., Ehrlich, S. & Krieg, H. A consistent and accurate ab initio parametrization of density functional dispersion correction (DFT-D) for the 94 elements H-Pu. *J. Chem. Phys.* **132**, 154104 (2010).

16 Stewart, J. J. P. *MOPAC2016*, HTTP://OpenMOPAC.net/ (2016).

17 Gershberg, J., Fennel, F., Rehm, T. H., Lochbrunner, S. & Würthner, F. Anti-cooperative supramolecular polymerization: a new K_2–K model applied to the self-assembly of perylene bisimide dye proceeding via well-defined hydrogen-bonded dimers. *Chem. Sci.* **7**, 1729-1737 (2016).
